# Desmoglein compensation hypothesis fidelity assessment in Pemphigus

**DOI:** 10.3389/fimmu.2022.969278

**Published:** 2022-09-23

**Authors:** Lauren Sielski, John Baker, Michael C. DePasquale, Kristopher Attwood, Kristina Seiffert-Sinha, Animesh A. Sinha

**Affiliations:** ^1^ Department of Dermatology, Jacobs School of Medicine and Biomedical Sciences, University at Buffalo, Buffalo, NY, United States; ^2^ Department of Biostatistics and Bioinformatics, Roswell Park Cancer Institute, Buffalo, NY, United States

**Keywords:** desmoglein compensation hypothesis, pemphigus, autoantibodies, morphology, fidelity

## Abstract

The pemphigus group of autoimmune blistering diseases encompasses pemphigus vulgaris (PV) and pemphigus foliaceus (PF). Lesion location in pemphigus has been elegantly postulated by the Desmoglein Compensation Hypothesis (DCH), which references the distribution of desmoglein (Dsg) proteins in the epidermis along with a patient’s autoantibody profile to describe three different lesion phenotypes: PF is characterized by subcorneal lesions in the presence of anti-Dsg1 antibodies only, while lesions in PV are suprabasilar and accompanied by anti-Dsg3 antibodies only in mucosal PV, or both anti-Dsg3 and anti-Dsg1 in the case of mucocutaneous PV. While the validity of this hypothesis has been supported by several studies and is prominently featured in textbooks of dermatology, a number of logical inconsistencies have been noted and exceptions have been published in several small-scale studies. We sought to comprehensively assess the extent to which patient clinical and autoantibody profiles contradict the DCH, and characterize these contradictions in a large sample size of 266 pemphigus patients. Remarkably, we find that roughly half of active PV and PF patients surveyed present with a combination of lesion morphology and anti-Dsg3/1 levels that contradict the DCH, including: patients with a cutaneous only PV presentation, mucocutaneous disease in the absence of either Dsg3, Dsg1, or both, and mucosal disease in the absence of Dsg3 or presence of Dsg1. We also find stark differences in fidelity to the DCH based on ethnicity and HLA-association, with the lowest proportion of adherence in previously understudied populations. These findings underscore the need to expand our understanding of pemphigus morphology beyond the DCH, in particular for populations that have not been a focus in previous investigation.

## Introduction

Pemphigus is a group of rare autoimmune skin blistering diseases characterized by mucosal or oral lesions due to the presence of autoantibodies against desmosomal cadherin proteins involved in cell-adhesion. The Desmoglein Compensation Hypothesis (DCH) is an elegant theory first proposed by Stanley and Amagai that correlates clinical presentation of pemphigus with the profile of autoantibodies directed against the cadherins desmoglein (Dsg)3 and -1 as the drivers of site-specific loss of cell-cell adhesion and blister formation (1, 2). The DHC references the distribution and expression of Dsg3 and -1 proteins within epidermal tissues to explain lesion site (cutaneous vs. mucosal) and lesion depth (suprabasal vs. subcorneal). Dsg1 is found in higher concentrations towards the superficial layers of skin or mucosa, while Dsg3 is found in higher concentrations towards the basal layers of skin or mucosa. Non-mucosal skin has a higher expression of Dsg1 throughout the epithelium, while Dsg3 is concentrated in the basal epithelium. Conversely, the mucosa has a greater expression of Dsg3 throughout the epithelium, while Dsg1 is only expressed in the superficial epithelium. Based on this distribution pattern of the Dsg3 and Dsg1 proteins, the DCH postulates 3 subtypes of pemphigus: Pemphigus foliaceus (PF), mucosal-limited Pemphigus vulgaris (PV), or mucocutaneous PV (3).

In this framework, PF is characterized by the presence of anti-Dsg1 antibodies only and presents with subcorneal skin ulcerations on cutaneous surfaces only, as the high concentration of Dsg3 in the mucosa is thought to compensate for the lack of functional Dsg1. PV, on the other hand presents with deeper, suprabasal blister formation due to the presence of anti-Dsg3 antibodies. The mucosal-limited subtype of PV is characterized by the presence of anti-Dsg3 alone, as the suprabasilar Dsg1 in the skin compensates for the lack of Dsg3. The mucocutaneous subtype of PV is characterized by both anti-Dsg3 and anti-Dsg1 antibodies and presents with suprabasilar mucosal and cutaneous lesions, since neither Dsg3 or Dsg1 are able to compensate for the inactivation of the other.

While many studies support the validity of the hypothesis (1, 3–5) and it is prominently featured in textbooks of Dermatology, numerous researchers have pointed out discrepancies in the theory. These contradictions include the presence of anti-Dsg antibodies in the absence of skin or mucosal lesions (i.e. clinical remission) (6, 7), or conversely, the absence of anti-Dsg antibodies in active disease (8, 9). They also include discrepancies between lesion location and anti-Dsg3/1 profiles, such as the presence of either anti-Dsg3 or -1 antibodies in mucocutaneous disease (9), elevated anti-Dsg1 levels without anti-Dsg3 antibodies in mucosal PV (10, 11), and the presence of only suprabasilar cutaneous lesions without mucosal lesions (11–13). Importantly, the DCH also cannot account for the clinical observation that PV patients with elevated anti-Dsg 1 that do not develop subcorneal blisters, as might be predicted, and as PF patients do, despite having the necessary antibody correlated with this level of intraepidermal split (7).

Though numerous previous studies have noted patients whose clinical presentation and antibody profiles contradict the DCH, these studies were primarily case studies with a limited patient population, review papers, or posed questions regarding the DCH in the context of other experiments (6, 7, 9–13). Here, in a larger patient sample size consisting of 253 PV and 13 PF patients, some with longitudinal sampling dates, we sought to determine (a) how often patients’ phenotype and antibody profile contradict the postulates of the DCH, (b) in which way these patients’ data contradict the DCH, and (c) if there are additional demographic or genetic factors that affect DCH conformity. We present evidence indicating that while the DCH can explain approximately 50% of PV phenotypes, it does not account for the clinical presentation in the other half of cases, making a strong case for the need to modify/expand the hypothesis.

## Materials and methods

### Patient population

Patients were recruited from the Dermatology outpatient clinics at the University at Buffalo (IRB 456887), Michigan State University (IRB 05-1034) and Weill Cornell Medical College (IRB 0998-398), in addition to annual meetings of the International Pemphigus and Pemphigoid Foundation (IPPF) between 2001 and 2018. A written informed consent from every patient was obtained at the time of enrollment. At all visits, patients were seen in person by medical staff with extensive experience in assessing Pemphigus based on histological, clinical and serological criteria.

For all PV and PF patients included in the study, disease diagnosis was verified using established clinical and histopathologic criteria. Specifically, the diagnosis of PV and PF was determined by histopathological findings (suprabasilar acantholysis vs. subcorneal acantholysis, respectively) and DIF (IgG and C3 deposition in intercellular epidermis). Patients were also asked to provide information regarding their demographics, disease course, medical history, and family history. Current lesion location was assessed at the time of intake and patients were classified as having mucosal only-, mucocutaneous-, or cutaneous only lesions. Subsequently, venous blood samples were obtained, and serum was isolated *via* centrifugation and stored at -80°C for future analysis. Patients with multiple visits had venous blood and clinical information taken at all visits when possible. The maximum number of repeat samples from a single patient was 6, and the average number was 1.4 samples per patient. Additionally, 221 healthy controls were enrolled, including 58 individuals with and 163 without a family history of pemphigus. All study procedures were identical between controls, PV patients, and PF patients.

For this study, we enrolled 253 PV patients, 13 PF patients, and 221 healthy control subjects. As patients could present multiple times and in different phases of disease, we collected 159 samples from 142 PV patients in the active phase of disease, 235 samples from 146 patients in remission, and 246 samples from 221 healthy control subjects. PF patient all presented in the active phase of disease. Our study population demographic data is summarized in **Supplemental Table 1**. Disease activity was defined for each patient using consensus guidelines developed by the International Pemphigus Committee (14). Patients were deemed to be active if they had three or more non-transient lesions (lasting more than 1 week) and/or extension of existing lesions. Patients were considered to be in complete remission if they experienced an absence of new or established lesions for at least 2 months. In a modification from the consensus guidelines, patients with transient lesions only (lasting less than 1 week) were classified as being in partial remission. In addition, patients in remission were assigned to one of two groups depending on the length of time they maintained clinical remission: newly remittent (2-6 months) and long-term remittent (>6 months). We refer to this expanded clinical subgrouping as “disease phase.” Information used to assign disease phase classifications was obtained from clinical assessment at the time of the visit and supplemented by patient history. At each study visit, patients were asked detailed questions about the time course of their lesions to assist in classification.

Therapy regimens in PV and PF vary considerably for each patient. To simplify, each patient was assigned a therapy status based on consensus guidelines (14) at each blood draw. “Minimal” therapy was defined by prednisone doses of ≤10mg/day and/or minimal adjuvant therapy for at least 2 months. Patients receiving >10mg/day of prednisone, IVIg, cyclosporin, dapsone, rituximab or other biologic agents were defined as “more than minimal” therapy. “Off” therapy was reserved for patients that were not receiving any systemic therapy. Our group has previously shown that therapy status defined as above does not significantly affect anti-Dsg levels in clinically active patients (6). Nevertheless, in order to reassess a potential effect of this extrinsic variable on autoantibody levels in our larger patient population, we determined anti-Dsg3 and -1 levels in patients classified according to disease activity and treatment status in this study population. We did not see any significant differences between anti-Dsg3/1 levels and treatment status in PV subjects in any phase of disease activity with the exception of patients in partial remission where anti-Dsg3 levels were found to be higher in the more than minimal group compared to the off-therapy group. (p = 0.02) (**Supplementary Figures 1A-C**). Thus, the analyses presented in this manuscript include patients on more than minimal, minimal and off therapy.

### Detection of anti-desmoglein 3 and 1 levels

Anti-Dsg ELISA was performed *via* standard protocols using Dsg 3 and Dsg 1 test systems by MBL Intl. (RG-M7593-D) with a 1:101 serum dilution (or 1:1000 in a limited number of samples with antibody levels over 140 IU/ml at 1:101 dilutions, which were considered too high for accurate detection at that dilution). The kits detect immunoglobulin G (IgG) antibodies against Dsg 3 and Dsg 1 and do not distinguish between subclass of IgG. Antibody positivity was defined at three separate ELISA levels of >36/37 IU/mL, >20 IU/mL, and >10 IU/mL for both anti-Dsg3 and anti-Dsg1. The >36/37 IU/mL cutoff was as per current manufacturer recommendations, while the >20 IU/mL cutoff was recommended by the manufacturer prior to 10/31/14. However, from years of experience using these ELISA kits, we felt that both these cut-offs are too stringent and exclude patients with lower antibody levels that are still clinically relevant. Thus, we determined the mean and standard deviation of anti-Dsg3 and anti-Dsg1 levels amongst healthy controls that did not carry the pemphigus associated HLA alleles DRB1*0402 and DQB1*0503 and had no family history of disease (n=96, mean anti-Dsg3 levels = 1.64 ± 4.57 IU/mL, mean anti-Dsg1 levels = 1.97 ± 4.12 IU/mL). These individuals were excluded to eliminate the possibility of elevated anti-Dsg3 and -1 levels in genetically susceptible but healthy individuals that might skew the determination of the threshold for anti-Dsg3 and -Dsg 1 positivity. We additionally excluded control subjects with family history of disease to also control for the presence of rarer PV-associated alleles in the control population that may similarly lead to elevated autoantibody values in healthy individuals. We then added two standard deviations to each mean (anti-Dsg3: 10.78 IU/mL, anti-Dsg1: 10.21 IU/mL) to establish a lower cutoff of 10 IU/ml to be used in addition to the manufacturer recommended levels. We present our data for each cut off separately.

### Detection of anti-thyroid peroxidase levels

Anti-thyroid peroxidase (TPO) levels were detected by ELISA as per manufacturer’s recommendation (GenWay Biotech, GWB-521202). The kit detects immunoglobulin G (IgG) antibodies against TPO and does not distinguish between subclasses of IgG. Antibody positivity was defined as >20 IU/ml. Given that the frequency of anti-TPO antibody positivity in healthy euthyroid individuals has been estimated to be around 8% (15), this cutoff was determined from referencing other studys’ cutoffs for anti-TPO (16), and choosing the one that resulted in 7.6% of our control population being positive for anti-TPO.

### Detection of anti-thyroglobulin levels

Anti-thyroglobulin (Tg) levels were detected by ELISA as per manufacturer’s recommendation (GenWay Biotech, GWB-521201). The kit detects immunoglobulin G (IgG) antibodies against Tg and does not distinguish between subclasses of IgG. Antibody positivity was defined as >5 IU/mL by first determining the mean and standard deviation of anti-Tg levels amongst healthy controls (1.18 ± 2.03 IU/mL) and then adding two standard deviations to the mean (5.24 IU/mL).

### HLA typing

High resolution HLA typing was performed by PCR amplification with sequence specific primers (17, 18) at the Histocompatibility and Immunogenetics Laboratory at Michigan State University using commercial kits (One lambda, Thermo Fisher Scientific). “HLA-positivity” (HLA^+^) was defined as the presence of one or both of the PV-associated HLA alleles, DRB1*0402 and DQB1*0503 (19). Patients not carrying either of these alleles were labeled as “HLA-negative” (HLA^-^).

### Statistical analysis

In order to assess variance in the anti-Dsg3/1 ELISA levels across disease activities (active, partial remission, complete remission) and vs. control subjects and across treatment status (more than minimal, minimal, and off therapy) we used Kruskal-Wallis testing. The Kruskal-Wallis test was initially performed across all subgroups for disease activity and treatment status. If the broader testing of all subgroups resulted in a value of p ≤ 0.05, further analysis of each individual subgroup was completed. P-values of ≤ 0.05 were considered to be significant.

In order to elucidate disease modifying factors, we compared the proportion of contradiction to the DCH among different ethnicities, HLA status, anti-TPO or anti-Tg positivity/negativity using chi-squared analyses. The proportion of DCH conformity and contradiction in the Ashkenazi Jewish population was compared to that of each of the remaining ethnicities, as this group had the highest rate of conformity and was the classic group studied in pemphigus. For HLA status, the percentages of conformity and contradiction were compared between subjects carrying the PV-associated HLA alleles DRB1*0402 and/or DQB1*0503 (“HLA-positive”) and those that did not carry the aforementioned alleles (“HLA-negative”). We also compared the proportion of DCH conformity and contradiction among anti-TPO or -Tg positive, anti-TPO or -Tg negative, and all PV subjects. P-values of ≤ 0.05 were considered to be significant.

## Results

### Both anti-Dsg3 and anti-Dsg1 antibodies can be detected in patients in disease remission

Numerous studies have shown that anti-Dsg antibody levels rise and fall in parallel to levels of disease activity (20, 21; K. E. 22; V. K. 23). However, it has also been noted that anti-Dsg1 and particularly anti-Dsg3 levels can remain elevated in states of disease remission (K.E. 6, 24). In order to assess the degree to which antibody profiles adhere to the postulate that lesions only appear in the presence of anti-Dsg3 and -1 antibodies in our PV patient population (n=253), we determined the proportion of patients that had positive anti-Dsg3 and -1 levels in active disease, in partial remission, and in complete remission. Anti-Dsg3 and -1 positivity was determined using three different cutoffs, two recommended by the manufacturer at different time points and one calculated in our laboratory based on comparison with a large set of healthy volunteers (see Materials and Methods). We show that among PV subjects that were clinically active (n=159), 77.99% were anti-Dsg3^+^, and 34.59% were anti-Dsg1^+^. However, despite exhibiting active disease, 18.24% carried neither anti-Dsg3 nor anti-Dsg1 autoantibodies when using the positivity cutoff of 20 IU/mL (see Materials and Methods) (similar percentages were seen across all cutoffs, **Table 1**), indicating that anti-Dsg3/1 antibodies are not the sole drivers of lesional activity. Depending on the cut-off value chosen, in PV subjects that were clinically in partial remission (i.e. presence of transient lesions only), 59.68-66.13% subjects were anti-Dsg3^+^, and 16.13-24.19% were anti-Dsg1^+^. Among PV patients that were clinically in complete remission, 7.30-15.73% were still anti-Dsg1^+^ and 44.38-61.24% were still anti-Dsg3^+^ (**Table 1**). As expected, in the healthy control population (n=246), anti-Dsg3 and anti-Dsg1 positivity was low to undetectable (anti-Dsg3 with a 0.40-1.59% positivity rate; anti-Dsg1 with a 0.79-4.37% positivity rate). Consistent with a previous study by our group performed in a slightly smaller patient cohort (197 PV patients), mean anti-Dsg1 levels significantly decreased from active to complete remission (p < 0.001) to levels below the threshold set by most studies for positivity (i.e. 20 IU/mL), while mean anti-Dsg3 levels decreased significantly from active to complete remission (p < 0.001), but often remained highly elevated even in remission (**Supplementary Figure 2** **).**

Table 1Presence of anti-Dsg3/1 antibodies in patients of varying states of disease activity.

**Table:** 

A. Anti-Dsg1.	Active (n = 159)	Partial remission (n = 62)	Complete remission (n = 178)	Control (n = 246)
Anti-Dsg1 Median (IQR) (IU/ml)	5.88 (48.81)	2.92 (7.80)	2.11 (5.42)	1.28 (2.78)
Anti-Dsg1^+^ patients (cut off >36 IU/ml)	30.19%	16.13%	7.30%	0.81%
Anti-Dsg1^+^ patients (cut off >20 IU/ml)	34.59%	17.74%	8.99%	2.44%
Anti-Dsg1^+^ patients (cut off >10 IU/ml)	40.25%	24.19%	15.73%	4.07%

**Table:** 

B. Anti-Dsg3.	Active (n=159)	Partial remission (n = 62)	Complete remission (n = 178)	Control (n = 246)
Anti-Dsg3 Median (IQR) (IU/ml)	105.5 (128.50)	76.2 (126.87)	24.47 (107.22)	0.61 (1.67)
Anti-Dsg3^+^ patients (cut off >37 IU/ml)	74.84%	59.68%	44.38%	0.41%
Anti-Dsg3^+^ patients (cut off >20 IU/ml)	77.99%	59.68%	51.12%	0.81%
Anti-Dsg3^+^ patients (cut off >10 IU/ml)	81.76%	66.13%	61.24%	2.03%

IU, International Units; Dsg, desmoglein.

Antibodies are presented as median and interquartile range (IQR) as well as percent positive at different cut-off levels.

### Anti-Dsg antibody positivity and lesion location do not follow the phenotype predicted by the DCH in more than half of active PV patients

In order to determine the degree to which the disease phenotype of active patients follows the postulates of the DCH, we compared the patient’s lesion location and corresponding anti-Dsg3 and -1 levels at the time of blood sampling. In order to conform to the postulates of the DCH, PF patients would be expected to only have detectable anti-Dsg1 antibodies, mucosal PV patients should only have detectable anti-Dsg3 antibodies, and mucocutaneous PV would be expected to harbor both anti-Dsg3 and anti-Dsg1 antibodies. Of note, the presence of patients with cutaneous PV (cPV), i.e. presence of suprabasal acantholysis limited to cutaneous lesions, inherently does not follow the DCH.

**Figure 1:**
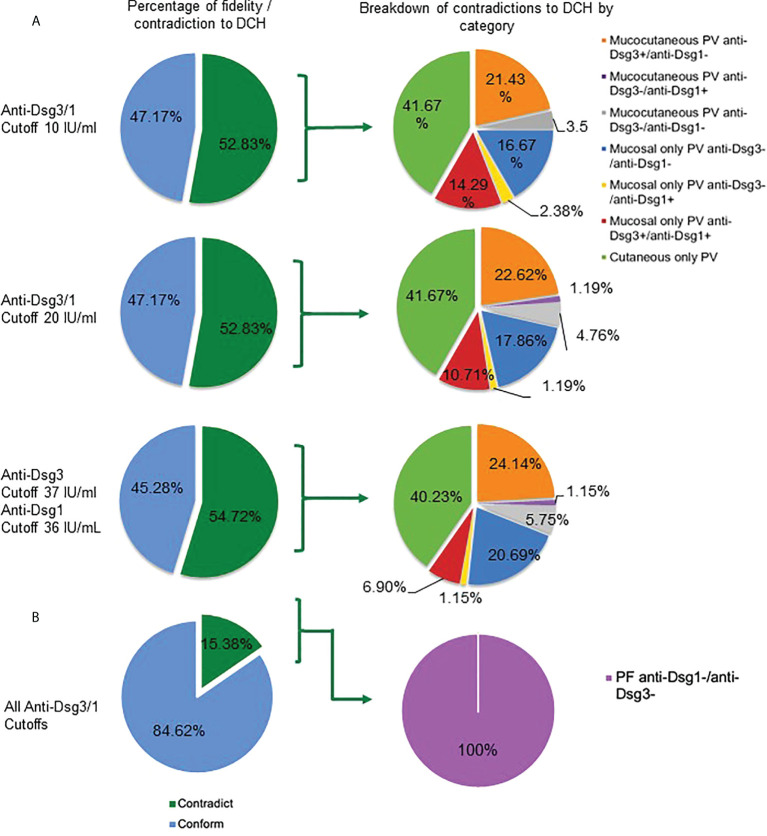
Contradictions to the DCH are consistent between different antibody cutoff values. **(A)** In active PV subjects (n = 159), more than half contradict the DCH based on their clinical lesions and autoantibody profile across all cutoffs for anti-Dsg3 and -1 positivity (10 IU/mL, 52.83%; 20 IU/mL, 52.83%; 37&36 IU/mL, 54.72%) (dark green shading in left column at a cut-off of 10 IU/ml, 20 IU/ml, and 36/37 IU/ml). Subjects that contradict the DCH were classified into seven categories based on how a PV subject may contradict the DCH (right column): i) mucocutaneous PV in the presence of the following antibody patterns: a) anti-Dsg1-/3+, b) anti-Dsg1+/3-, and c) anti-Dsg1-/3-, ii) mucosal only PV carrying a) anti-Dsg1-/3-, b) anti-Dsg1+/3-, and c) anti-Dsg1+/3+, and iii) cutaneous only PV (with any antibody pattern). **(B)** For active PF subjects (n = 13), we found that 15.38% across all cutoffs do not follow the DCH according to their lesion location and anti-Dsg1 and -3 positivity at the same visit. The only contradiction seen in the PF group was the absence of both anti-Dsg1 and anti-Dsg3.

Surprisingly, we found that for active PV patients visits (n=159), over 50% of the subjects displayed lesion morphology and corresponding anti-Dsg3 and -1 profiles that contradict the postulates of the DCH regardless of the cut-off value for antibody positivity chosen (ranging from 52.83% for the lowest cut-off of 10IU/ml to 54.72% for the cut-off of 36/37 IU/ml currently suggested by the manufacturer) (**Figure 1A**). In contrast, in the active PF patient group (n=13), only 15.38% of patients were found to contradict the DCH (**Figure 1B**).

### Among multiple observed contradictions to the DCH, the presence of “cutaneous only” PV is the most common

To define the extent and range of contradictions to the DCH, we differentiated multiple clinical subgroups based on a combination of morphology and anti-Dsg3 and -1 antibody levels. We found several categories of patients that would not be predicted to occur according to the DCH. We observed eight permutations of contradictions to the DCH within PV patients: i) mucocutaneous PV in the presence of the following antibody patterns: a) anti-Dsg3+/1-, b) anti-Dsg3-/1+, and c) anti-Dsg3-/1-, ii) mucosal only PV carrying a) anti-Dsg3-/1-, b) anti-Dsg3-/1+, or c) anti-Dsg3+/1+, iii) cutaneous only PV (with any autoantibody pattern), and iv) a PF phenotype in the absence of anti-Dsg3 and -1 (**Figure 1**).

Among these contradictions, the cutaneous PV clinical phenotype was found to be the most common violation of the DCH (41.67% using the 20 IU/mL cutoff), with 84 subjects presenting with cutaneous only lesions PV at the time of visit (**Figure 1**). Seven of the cutaneous only PV (cPV) subjects were identified without having had any *history of* mucosal lesions (cPVwohm). The anti-Dsg levels for cutaneous only PV subjects were approximately equally distributed across categories of patients carrying anti-Dsg3^+^/anti-Dsg1^+^, anti-Dsg3^-^/anti-Dsg1^+^, anti-Dsg3^+^/anti-Dsg1^-^, and anti-Dsg3^-^/anti-Dsg1^-^ antibody profiles (**Supplementary Figure 3**). Cutaneous only PV was followed in frequency in contradiction to the DCH by mucocutaneous PV in the presence of anti-Dsg3 antibodies only (22.62%), mucosal PV with anti-Dsg3^-^/anti-Dsg1^-^ (17.86%), mucosal PV with anti-Dsg3^+^/anti-Dsg1^+^ (10.71%), mucocutaneous with anti- Dsg3^-^/anti-Dsg1^-^ (4.76%), and equal numbers of mucocutaneous with anti-Dsg3^-^/anti-Dsg1^+^ (1.19%) or mucosal with anti-Dsg3^-^/anti-Dsg1^+^ (1.19%) (**Figure 1A**). The only contradiction type found in the PF population was the absence of both anti-Dsg3 and -1 antibodies (**Figure 1B**)

### Contradictions to the DCH are more prevalent in non-Ashkenazi Jewish ethnicities and in HLA negative PV subjects

**Figure 2:**
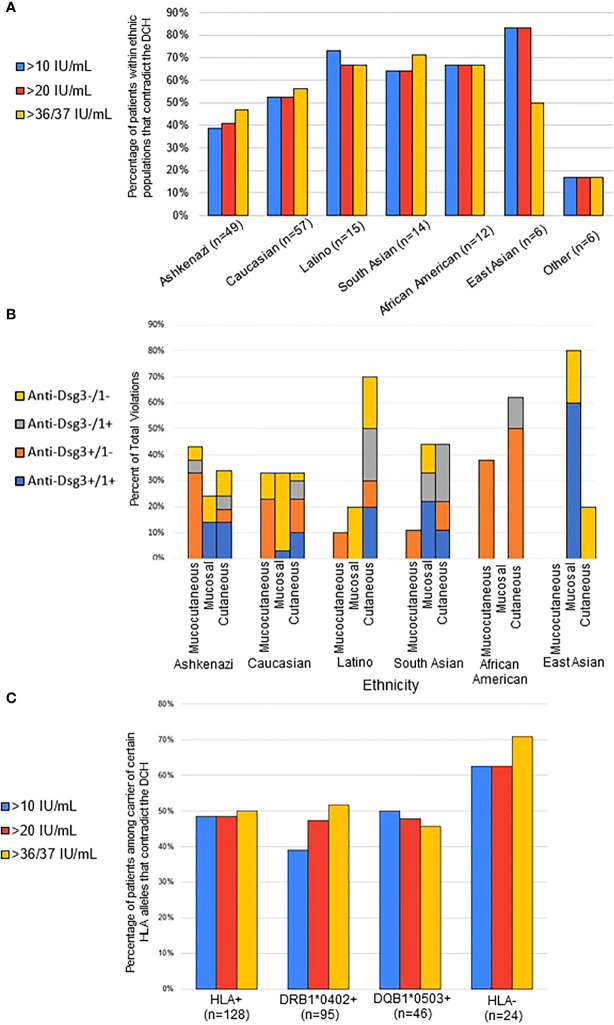
Contradictions to the DCH differ by ethnicity and HLA association. **(A)** For each ethnicity, the proportion of conformity and contradiction to the DCH was determined based on their lesion phenotype and anti-Dsg3/1 positivity for each visit. The percentage of contradiction is presented using the 10-, 20-, and 37/36 IU/mL anti-Dsg cutoff, with similar percentages seen in all cutoffs. The Ashkenazi Jewish population had a noticeably smaller proportion of subjects that contradicted the DCH compared to those of Caucasian, South Asian, African American, Latino, and East Asian ethnicities (p=0.09). **(B)** Within each ethnic group, the type of violation of the DHC was broken down into specific lesion morphology and associated anti-Dsg profile. Results are presented as percent of violation within the specific ethnic group. **(C)** Subjects were considered “HLA-positive” (HLA^+^) if they carried either of or both the previously described PV-associated susceptibility HLA alleles DRB1*0402 and/or DQB1*0503. All active PV patients were classified as either HLA^+^, only DQB1*0402-positive, only DQB1*0503-positive, or negative for either allele (HLA^-^). We found that 48.44% (n = 128) of those that are HLA+ contradict, 47.37% (n = 95) that are DRB0402^+^ contradict, 47.83% (n = 46) that are DQB0503^+^ contradict, and 62.50% (n = 24) that are HLA^-^ for PV alleles contradict (p=0.06 when using chi-square with the anti-Dsg3/1 cutoffs of 37 and 36 respectively).

It has been reported that clinical phenotypes as well as HLA distribution vary among PV patients of different ethnicities (24). In order to analyze whether ethnicity plays a role in a patient’s fidelity to the postulates of the DCH we sub-grouped all active PV subjects by ethnicity and determined the combination of disease phenotypes and anti-Dsg levels that conformed with the DCH (n=75, 20 IU/mL cutoff) to those that did not (n=84, 20 IU/mL cutoff). We found that the lowest proportion of contradictions to the DCH by ethnic group are observed in the Ashkenazi Jewish (40.8%, n=49) and Caucasian populations (52.6%, n=57). We see even greater deviation from the DCH in the Latino (66.7%, n=15), South Asian (64.3%, n=14), African-American (66.7%, n=12), and East Asian (83.3%, n=6) populations. In six patients defined as “other” (multiracial), only 16.7% (n = 6) contradicted the DHC (**Figure 2A**). Thus, the Ashkenazi Jewish population had a noticeably smaller proportion of subjects that contradicted the DCH compared to those of Caucasian, South Asian, African American, Latino, and East Asian ethnicities (p=0.09). While this difference just missed the threshold of statistical significance at p=0.05, it is possible that the small sample sizes of non-Ashkenazi and non-Caucasian ethnic groups are masking true significant relevance of population differences.

Given the different rates of violating the DCH between different ethnicities, we then analyzed the types of DCH violations displayed by different ethnic groups (**Figure 2B** **)**. Notably, we found that the two populations with the least contradictions of the DCH, Ashkenazi Jewish and Non-Jewish Caucasian, had the least variation in lesion morphology in DCH violations, where the other populations had bias towards specific lesion morphologies, particularly the cutaneous only phenotype for African Americans as well as Latinos. We also note that the African American population violations were overwhelmingly anti-Dsg3+/1-, regardless of lesion morphology, while over half of all violations in Caucasian patients presented with a double negative anti-Dsg3-/1- antibody profile.

The strong correlation between certain HLA types, particularly DRB1*0402 or DQB1*0503, and PV is well accepted (25). Thus, we also examined the relationship between HLA status and DCH adherence. The rate of contradiction to the DCH was determined for PV subjects that carried either: i) DRB1*0402, or DQB1*0503, or both alleles together (“HLA-positive”, HLA^+^), ii) only DRB1*0402, iii) only DQB1*0503, or iv) neither of these alleles (“HLA-negative^”,^ HLA^-^). Approximately half of all active PV patients that carry at least one of the PV-associated HLA susceptibility alleles follow the postulates of the DCH. On the other hand, a higher number (62.5%) of active PV patients that are “HLA-negative “violate the DCH compared to “HLA-positive” patients (p=0.06 when using chi-square with the anti-Dsg3/1 cutoffs of 37 & 36 respectively) (**Figure 2C**).

### Autoantibody profiles differ by ethnicity

**Figure 3:**
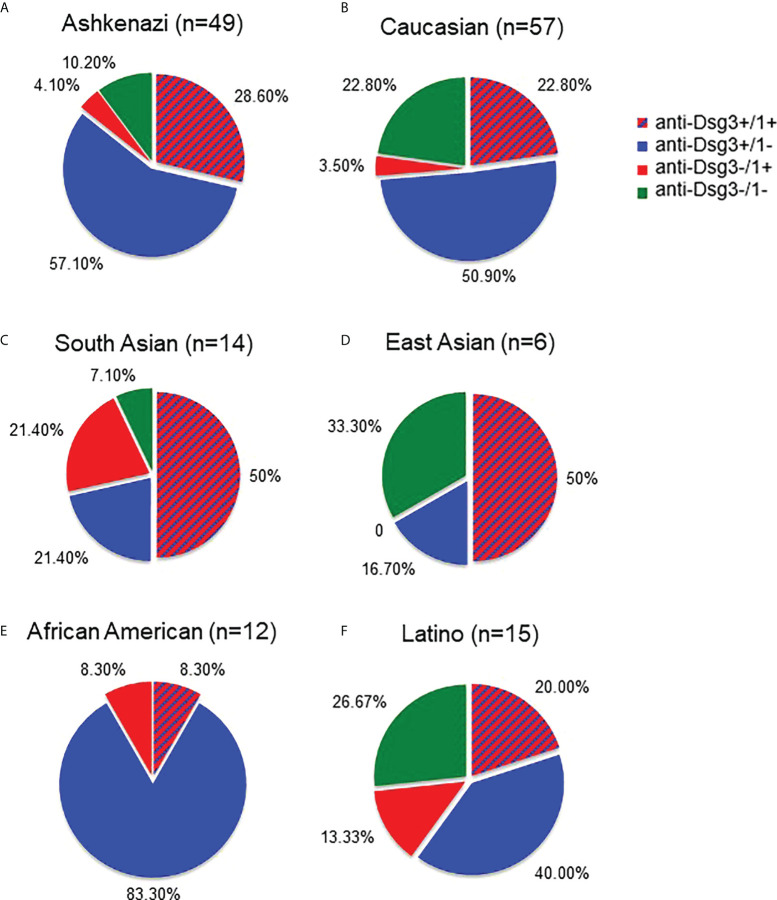
Autoantibody profiles differ between Ashkenazi and non-Ashkenazi ethnicities. We compared the anti-Dsg3/1 autoantibody profiles of all active PV subjects according to their self-identified ethnicity. The Ashkenazi Jewish population **(A)** was found to have a predominantly anti-Dsg3^+^/1^-^ profile. The Caucasian population **(B)** showed a similar profile to that seen in the Ashkenazi ethnicity, though with slightly less anti-Dsg3^+^/1^-^ and greater anti-Dsg3^-^/1^-^ percentages. The South Asian **(C)** antibody profile showed a greater proportion of anti-Dsg3^-^/1^+^ (21.4%) with a greater total percentage of total anti-Dsg1^+^ (71.4%) than seen in the Ashkenazi and Caucasian populations (32.7% and 26.3%, respectively). Additionally, the East Asian population **(D)** shows predominantly anti-Dsg3^+^/1^+^ and anti-Dsg3^-^/1^-^ patterns. The African American population **(E)** shows a largely anti-Dsg1^-^/3^+^ profile. The Latino population **(F)** shows a relatively even distribution across anti-Dsg patterns, with slightly more anti-Dsg3^+^/1^-^ seen.

Given that we found a higher proportion of contradictions to the DCH in non-Ashkenazi ethnicities compared to the Ashkenazi Jewish PV population, we wanted to assess whether autoantibody profiles of each ethnicity in our study population differed as well. We examined all active PV subjects according to their ethnicity, regardless of whether they contradict or conform to the DCH, and classified their autoantibody profile from each visit’s blood draw into one of four subgroups; i) anti-Dsg1^+^/3^+^, ii) anti-Dsg1^-^/3^+^, iii) anti-Dsg1^+^/3^-^, and iv) anti-Dsg1^-^/3^-^, using the 20 IU/mL cutoff for anti-Dsg1/3 positivity. Similar to a previous study by Harman et al. (24), we found that South Asian PV subjects showed a greater proportion of anti-Dsg1 positivity (n=14, 71.4%) compared to Ashkenazi Jewish (n=49, 32.5%) and Caucasian (n=57, 26.3%) groups (**Figures 3A–C**). Conversely, of these 3 populations, Ashkenazi Jewish patients carry the highest percentage of anti-Dsg3 antibodies (85.7%, **Figure 3A**). The overall anti-Dsg3 and -1 profile in the Latino population appears similar to that of the Caucasian population, with slightly more anti-Dsg1^+^/3^-^ and less anti-Dsg1^-^/3^+^ (**Figure 3F**). Interestingly, African American patients show the highest percentage of anti-Dsg3 positivity (**Figure 3E**). The interpretation of these data is limited by the small sample sizes, particularly in the East Asian population (**Figure 3D**).

### Subjects with detectable levels of anti-thyroid peroxidase and anti-thyroglobulin antibodies show a greater percentage of contradiction to the DCH than subjects not carrying these antibodies

**Figure 4:**
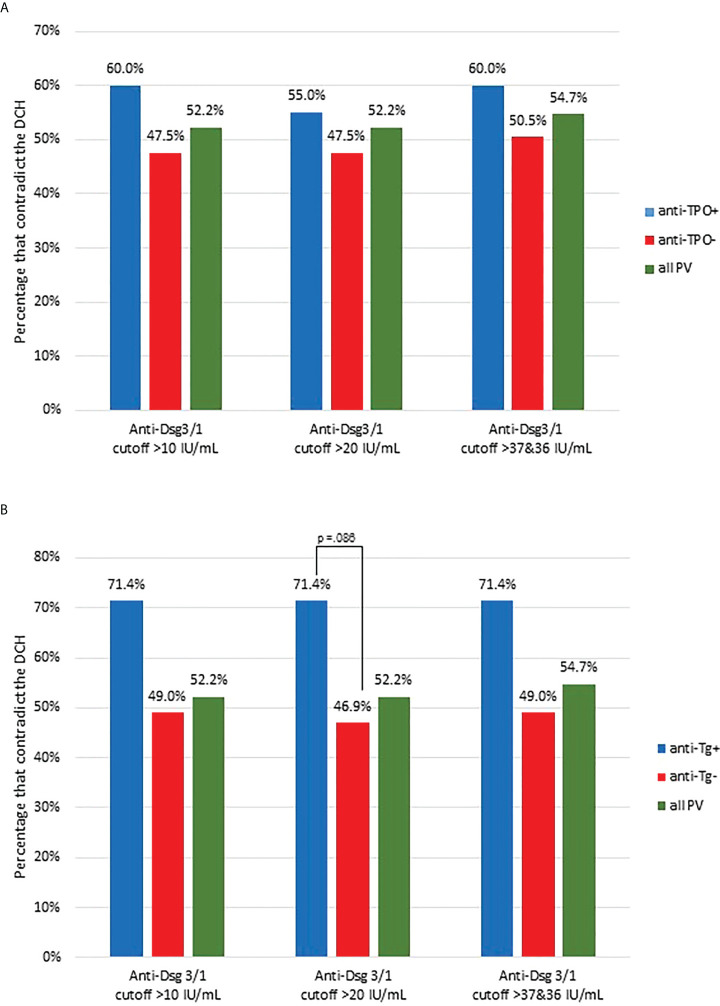
Proportion of contradictions to the DCH are slightly increased in PV patients carrying thyroid disease associated antibodies. **(A)** The proportion of conformity and contradiction to the DCH was assessed in all active PV subjects that had positive anti-TPO antibodies (anti-TPO+, n=20) and compared this proportion to those with negative anti-TPO levels (anti-TPO-, n=101) and all active PV subjects (all PV, n=159). The percentage of contradiction is presented using the 10-, 20-, and 36/36 IU/mL cutoff for anti-Dsg1/3. **(B)** Anti-Tg+ PV subjects had a higher proportion of contradiction to the DCH than anti-Tg^-^ and all PV subjects. We assessed the proportion of conformity and contradiction in all active PV subjects that had positive anti-Tg positive antibodies (n=14) and compared this proportion to those with negative anti-Tg levels (n=96) and all active PV subjects (n=159). The percentage of contradiction is presented using the 10-, 20-, and 36/36 IU/mL cutoff for anti-Dsg1/3. The difference between anti-Tg+ and anti-Tg- subjects is trending towards significance (p=0.086).

Previous studies from our group have established that non-Dsg antibodies, such as anti-TPO and anti-Tg are found at elevated rates in PV patients, particularly in patients with no detectable levels of anti-Dsg3 and -1 antibodies (26). In order to investigate whether these antibodies play a discernable role in a patient’s adherence/non-adherence to the DCH, we analyzed subjects who were carriers of either anti-TPO or anti-Tg and compared their proportion of contradiction to the DCH with that of anti-TPO negative or anti-Tg negative, and all active PV subjects. We found a higher, albeit non-significant, percentage of contradiction among anti-TPO^+^ PV subjects (n=20) across all cutoffs compared to all anti-TPO^-^ subjects (n=101) and the entire active PV study population (n=159) (**Figure 4A** **)**. Similarly, a higher proportion of anti-Tg^+^ PV subjects (n=14) contradict the DCH than anti-Tg^-^ subjects (n=96) and all PV subjects (n=159) across all cutoffs (trending towards significance with Chi Square of p=0.086) **(** **Figure 4B** **)**. The breakdown of lesion location was approximately equal in the TPO+ population that contradicted the DCH (5 cutaneous, 5 mucocutaneous and 5 mucosal), while it skewed toward cutaneous presentation for the Tg+ population (5 cutaneous, 2 mucocutaneous and 3 mucosal). Of note, there was one PF subject that was anti-TPO^+^ and this subject contradicted the DCH across all cutoffs.

## Discussion

The Desmoglein Compensation Hypothesis is an elegant theory that attempts to explain lesion morphology of pemphigus patients based on anti-Dsg3/1 antibody profiles. Original studies underlying this hypothesis were based on the observation that autoantibodies in PV patients target intercellular adhesion molecules (1), and that patients with distinct clinical presentations (mucosal dominant PV, mucocutaneous PV, and PF) display different autoantibody profiles (2, 3). These findings along with the known distribution of desmoglein proteins within the epithelium led to the creation of the DCH. Early animal studies as well as human *in vitro* analyses lent support to the hypothesis (3, 4, 27–29) and led to the DCH becoming a widely accepted theory for pemphigus pathogenesis. However, cases of “atypical” pemphigus were soon noted in the literature.

There are several instances in which the DCH is not followed in PV/PF, including (i) the presence of anti-Dsg antibodies without clinical disease (30), (ii) the absence of anti-Dsg1 or -3 antibodies in active disease (8, 9, 31), (iii) cases in which there is a mismatch of anti-Dsg levels and lesion type according to DCH postulates (10, 32), and (iv) the presence of cutaneous only PV (11, 33). To expand on these case studies, we performed a systematic and comprehensive analysis of the fidelity of the DHC in our large patient repository associated with carefully annotated clinical and epidemiologic data. We were able to confirm the presence of all potential contradictions to the DCH seen in previous studies. We also confirmed previous data from our group (6) generated from a smaller subset of the data we presented here showing that while anti-Dsg3 and -1 levels generally decline with diminishing disease activity, a sizeable number of patients (7.30-15.73%) still show the presence of anti-Dsg1 antibodies in complete clinical remission, and about half of patients in remission continue to exhibit positive levels of anti-Dsg3 antibodies. On the other hand, we also observed clinically active patients with negative anti-Dsg3 and -1 levels in mucocutaneous disease, negative anti-Dsg3 levels in mucosal disease, and negative anti-Dsg1 levels in PF, all scenarios that do not conform to the postulates of the DCH. Strikingly, greater than 50% of visits overall in active PV subjects and approximately 15% of visits in active PF subjects were found to contradict the DCH based on their clinical lesions and anti-Dsg profile. Of note, nearly two decades ago the Bystryn group found strikingly similar discrepancies with only 50% of patients with exclusively mucosal lesions carrying anti-Dsg3, only 53% of patients with both skin and oral lesions carrying anti-Dsg3^+^/1^+^, and just 72% of patients with exclusively skin lesions carrying anti-Dsg1^+^ (32). In our study, patients contradicted the predicted lesion morphology in a variety of ways (summarized in **Figure 1** **)**, with the most prevalent contradiction being the cutaneous only manifestation in PV. A cutaneous only presentation of PV is in violation of the postulates of the DHC per definition, as suprabasilar acantholysis of the skin in the absence of mucosal lesions is not predicted by any autoantibody profile. **Table 2** summarizes the antibody patterns observed in patients enrolled in the study and contrasts the expected morphology predicted by the postulates of the DHC with their actual clinical presentations.

**Table 2:** Expected vs. observed lesion morphology based on antibody profile.

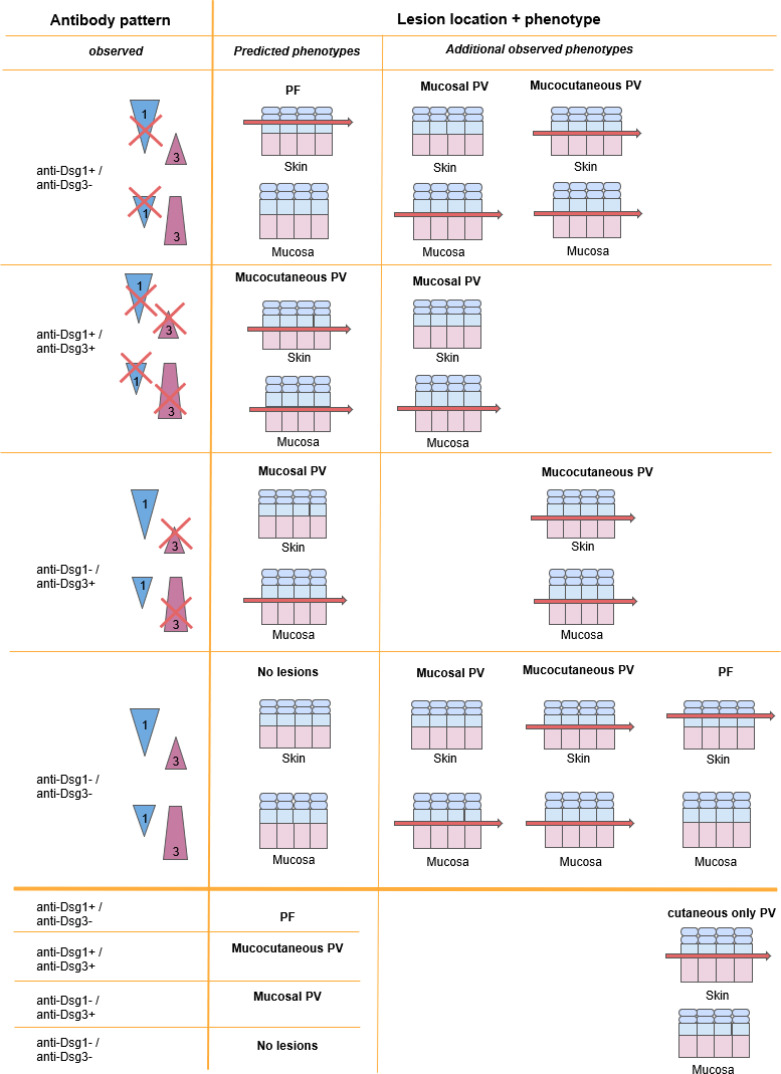

The left column displays the potential antibody combinations of anti-Dsg3 and anti-Dsg1 specificities in pemphigus patients. The expected phenotypes with a given antibody profile as expected per the DCH are noted in the middle column. The right column displays additional phenotypes observed in this study and by others that are not predicted by the DCH. Notably, cutaneous only PV, which is not predicted by the DCH, was seen with every combination of autoantibodies.

One possible limitation of this study is the use of ELISA for quantification of anti-Dsg antibodies. While ELISA has been the standard of anti-Dsg antibody detection for over 20 years, it has been found that ELISA kits coated with baculovirus-made Dsg3 and -1 use recombinant molecules that contain both mature protein and immature proprotein (34, 35). This has raised the concern the binding of unrelated antibodies to Dsg-proprotein could lead to false positive detection of anti-Dsg3 or 1 antibodies, as has been shown for anti-Dsg1 in healthy controls and patients with the unrelated autoimmune disease thrombotic thrombocytopenic purpura (35). Conversely, it has been shown that custom made mature-Dsg3-only ELISA plates that were created by treating the baculovirus-made recombinant Dsg3 with furin to convert Dsg3 pro-peptide to mature Dsg3 resulted in increased mean serum index values for anti-Dsg3 when compared to plates coated with baculovirus-made recombinant Dsg3 ELISA plates (34). However, no difference in diagnostic results was found when comparing these two methodologies (34). Consequently, in 2009, the manufacturer of the most commonly used anti-Dsg3/1 ELISA kit (MBL International), the system that was also used in this study, began treating baculoviral-expressed Dsg3 and Dsg1 with furin to increase the ratio of mature protein to proprotein on their ELISA plates. In September 2013, MBL International switched to Dsg proteins made in mammalian systems with CHO cells with highly efficient post-translational modification systems, thus reducing the presence of proproteins (personal communication). In any case, the early studies that supported the DCH in fact used baculovirus-made recombinant Dsg to detect anti-Dsg antibodies, generating clear patterns in patient antibody-profiles in relation to lesion morphology that were used to develop the DCH (2–4). If the presence of proprotein would be altering antibody reactivity in the work presented in our study, this would have also been the case in the early studies used to create the DCH. Another potential limitation of this study is that we do not have concomitant DIF/IIF data for the sample time points presented. The phenomenon of DIF/IIF positivity with negative serum antibody ELISA is a theoretical (but unlikely) possibility, and this scenario in itself would further bring into question the validity of the DCH as it would support the notion of non-Dsg antibodies as contributors to lesional activity.

There is the theoretical possibility that a given non-DCH conforming patient could present at an undetermined later time point with different lesion locations (mucosal only vs. mucocutaneous vs. cutaneous only) that render the patient conforming. However, the DCH by definition was formulated to explain current lesion location by matching with the current antibody profile, and the data presented in this manuscript clearly shows that there are limitations to this hypothesis. Potentially, future studies could be designed to include a well-defined (but unknown) follow-up period to assess the possibility of a transition to a DCH-conforming phenotype in patients previously non-conforming. In any case, the exact cut off allowing for a transitional time period would need to be determined and agreed upon, and any such cases would still command an adjustment to the DCH as currently framed.

Factors that have been proposed to help explain contradictions to the DCH include the differences in pathogenicity of autoantibodies (36), transient phenotypes (11), ethnicity (24), HLA status (37), and other non-Dsg autoantibodies in the context of the multipathogenic theory of pemphigus pathophysiology (8, 9, 37–39). We investigated three of these potential explanations, i.e whether contradictions occur at a higher rate according to ethnicity, HLA status, and the presence of other auto-antibodies such as anti-TPO and anti-Tg. Our study supports the assertion that ethnicity is one of the main drivers of autoantibody selection as well as adherence or non-adherence to the DCH, and also that DCH adherence is influenced by HLA type. The groups most likely to contradict the DCH included non-Ashkenazi Jewish ethnicities, populations not expressing the accepted PV-susceptibility alleles, and anti-Tg positive subjects. It is conceivable that many of the early studies leading to the formulation of the DCH were done in patients of Ashkenazi Jewish descent, as this ethnic group has one of the highest rates of PV (40) and comprises an overwhelming majority of Pemphigus patients not just in Israel, but also in the US. The higher DCH conformity rates in the Ashkenazi Jewish population as observed in our study could have skewed our understanding of disease towards a perspective from its presentation in this specific population, rather than that inclusive of broader and diverse ethnicities. Others have also noted that non-Ashkenazi populations, such as South Asians, exhibit an overall different anti-Dsg autoantibody profile than white ethnicities. In particular, South Asian PV patients have been reported as having a higher proportion of anti-Dsg1 positivity (24) and had a longer disease course than their white counterparts (41). Similarly, we found anti-Dsg profiles in South Asian subjects incorporated a higher percentage of anti-Dsg1 antibodies than their Ashkenazi Jewish counterparts, while Ashkenazi Jewish patients showed a higher percentage of anti-Dsg3 antibodies. Further studies with a larger sample size of previously underrepresented ethnicities are necessary to better elucidate these differences, especially in the East Asian and African American populations. Nonetheless, even among the Ashkenazi population, a large number of patients contradicted the postulates of the DHC, indicating that factors outside of ethnicity affect autoantibody selection and disease manifestation.

One of the factors that is related to, but also independent of ethnicity is the expression of the specific HLA alleles in a Pemphigus patient. The HLA system is a complex of genes that encodes the major histocompatibility complex in humans, the most polymorphic region of the human genome. Numerous HLA genes have been linked to autoimmune diseases; Pemphigus vulgaris has one of the strongest HLA associations of all autoimmune disease. Our group and others have shown that HLA genetic susceptibility in PV strongly, but not exclusively, maps to the HLA class II genes DRB1*0402 and DQB1*0503, with >80% of North American patients expressing one or both of these genes (19, 42). When comparing patients that are carriers of either one or both of these two alleles (termed “HLA^+^”) to patients that do not carry these alleles (termed “HLA^-^”), we found a higher proportion of DCH contradiction in the HLA^-^ group relative to the HLA^+^ group.

HLA molecules are a requirement for the initiation of disease *via* the presentation of as yet not clearly clarified (auto)antigens to CD4 cells that then prime B cells for autoantibody production (43, 44). Our data suggests that some of the patients who contradict the DCH carry HLA alleles that bind and present a different set of triggering (auto)antigens than the commonly PV associated HLA molecules DRB1*0402 an DQB1*0503. Recently, our group has shown that anti-TPO activity is heightened in the serum of North American PV patients and driven by HLA status as well as the absence of anti-Dsg3/1 reactivity (26). The presence of higher levels and rates of positivity of anti-TPO and anti-Tg antibodies in pemphigus patients compared to healthy controls was recently confirmed in the Chinese population (45). Interestingly, we found that both anti-TPO^+^ and anti-Tg^+^ subjects had a higher proportion of contradiction to the DCH compared to anti-TPO^-^ and anti-Tg^-^ subjects, respectively, suggesting that these antibodies may be involved in disease pathogenesis in certain cases not explained by the DCH, such as those not exhibiting detectable levels of anti-Dsg antibodies and/or PV in underrepresented HLA haplotypes.

Based on the accumulating evidence for the potential role of multiple non-Dsg autoantibodies in pemphigus pathogenesis, we further acknowledge that non-Dsg autoantibodies other than anti-TPO and anti-Tg could also be of relevance in cases that are nonadherent to the DCH. A number of groups have detected non-Dsg antibodies in the serum of PV patients including those directed against acetylcholine receptors, desmocollin, and mitochondria (9, 39, 44, 46, 47) that may provide an explanation of as to why patients with non-detectable anti-Dsg antibodies can nonetheless present in an active state of disease. Careful analysis of these autoantibodies and their impact on the expression and progression of clinical disease is an important area of future research.

Finally, there is also the possibility that antibody levels are not the only disease relevant factor in PV and that other, as yet undiscovered, genetic and/or skin structural factors contribute to disease presentation. Our results demonstrate a need to readjust the DCH as the standard explanation for disease expression in pemphigus, highlighting the complexity of auto-antibody involvement and clinical disease. Unraveling this complexity will be a required step in deepening our understanding of disease mechanisms and developing increasingly targeted and individualized therapies across the spectrum of pemphigus.

## Data availability statement

The raw data supporting the conclusions of this article will be made available by the authors, without undue reservation.

## Ethics statement

The studies involving human participants were reviewed and approved by Institutional Review Board, University at Buffalo; Institutional Review Board, Michigan State University; Institutional Review Board, Weill Medical College of Cornell University. The patients/participants provided their written informed consent to participate in this study.

## Author contributions

KS-S and AS devised the study and collected patient samples and associated clinical data; JB, LS, and MD performed the data analysis, JB, LS, KS-S, and AS drafted the article. KA provided statistical analysis of data. All authors contributed to the article and approved the submitted version.

## Acknowledgments

We acknowledge the many Pemphigus patients that have contributed their samples and time to this study.

## Conflict of interest

The authors declare that the research was conducted in the absence of any commercial or financial relationships that could be construed as a potential conflict of interest.

## Publisher’s note

All claims expressed in this article are solely those of the authors and do not necessarily represent those of their affiliated organizations, or those of the publisher, the editors and the reviewers. Any product that may be evaluated in this article, or claim that may be made by its manufacturer, is not guaranteed or endorsed by the publisher.

## Supplementary material

Supplementary Figure 1 Anti-Dsg Levels Across Treatment Status. Anti-Dsg3/1 levels in patients are shown based on based on level of therapy in active disease **(A)**, partial remission **(B)**, and complete remission **(C)**.

Supplementary Figure 2 Anti-Dsg Levels Across Disease Activity. Anti-Dsg1 levels **(A)** and anti-Dsg3 levels **(B)** are shown across varying levels of disease activity in patients as well as in the healthy control group.

Supplementary Figure 3 Contradictions in the DHC observed in active patients. The most common contradiction to the DCH observed in active PV is the cutaneous only variant of disease (cPV) (green shading in left column at a cut-off of 10 IU/ml, 20 IU/ml, and 36/37 IU/ml). A breakdown of contradictions within the cutaneous only manifestation of PV (right column) identifies all possible combinations of anti-Dsg3/Dsg1 expression.
