# Supplementary figures and images for: Desmoglein compensation hypothesis fidelity assessment in Pemphigus
Source: Front Immunol. 2022 Sep 23;13:969278. doi: 10.3389/fimmu.2022.969278 (PMC9537551; doi:10.3389/fimmu.2022.969278)

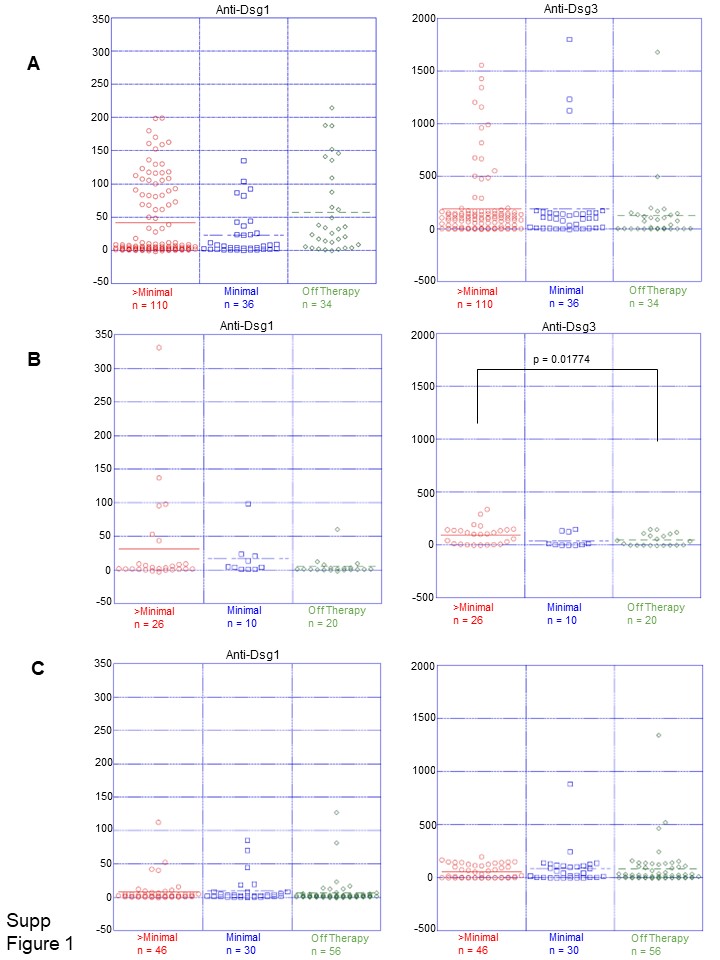

Supplement: Supplementary Figure 1 — Anti-Dsg Levels Across Treatment Status. Anti-Dsg3/1 levels in patients are shown based on based on level of therapy in active disease (A), partial remission (B), and complete remission (C). [file Image_1.jpeg]

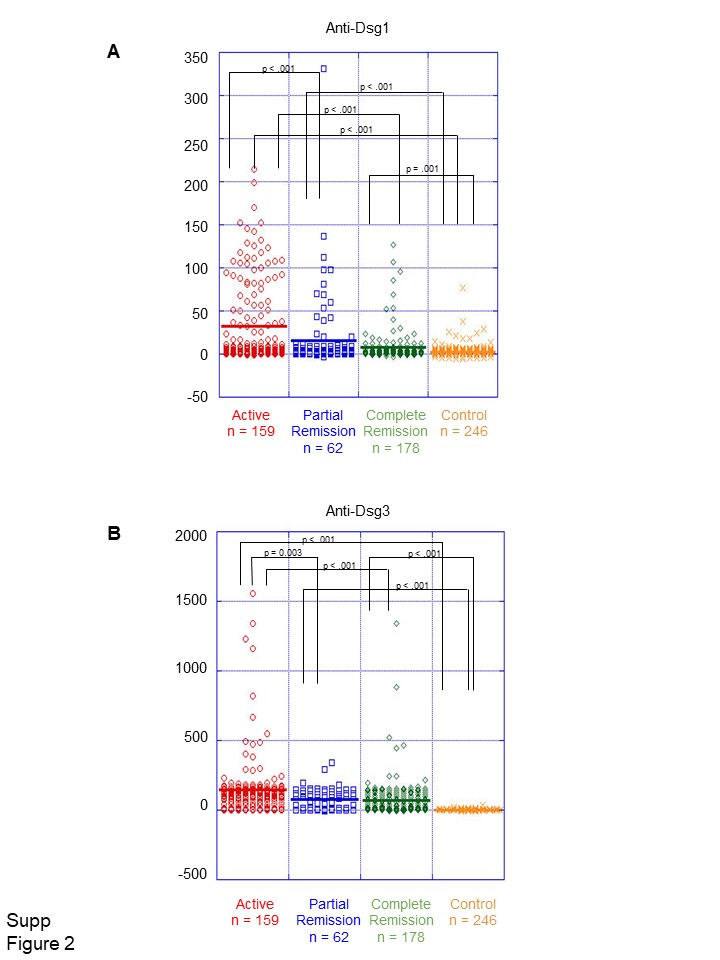

Supplement: Supplementary Figure 2 — Anti-Dsg Levels Across Disease Activity. Anti-Dsg1 levels (A) and anti-Dsg3 levels (B) are shown across varying levels of disease activity in patients as well as in the healthy control group. [file Image_2.jpeg]

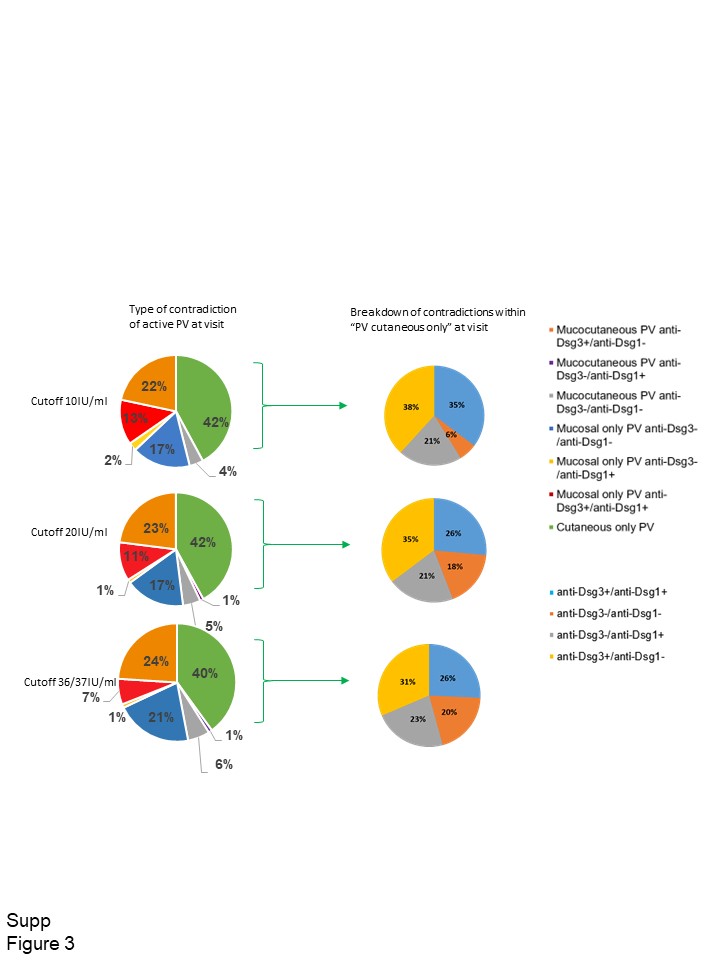

Supplement: Supplementary Figure 3 — Contradictions in the DHC observed in active patients. The most common contradiction to the DCH observed in active PV is the cutaneous only variant of disease (cPV) (green shading in left column at a cut-off of 10 IU/ml, 20 IU/ml, and 36/37 IU/ml). A breakdown of contradictions within the cutaneous only manifestation of PV (right column) identifies all possible combinations of anti-Dsg3/Dsg1 expression. [file Image_3.jpeg]
