# Supplementary material for: Desmoglein compensation hypothesis fidelity assessment in Pemphigus
Source: Front Immunol. 2022 Sep 23;13:969278. doi: 10.3389/fimmu.2022.969278 (PMC9537551; doi:10.3389/fimmu.2022.969278)
Supplement: Supplementary file 4 [file Table_1.docx]

|  | **PV Patients** | **PF Patients** | **Controls** |
| --- | --- | --- | --- |
| **Number of Subjects** | 253 | 13 | 221 |
| **Number of Samples** | 394 | 13 | 246 |
| **Male:Female Ratio** | 87:166 (1:1.91) | 6:7 (1:1.17) | 90:121 (1:1.34) |
| **Average Age** | 54.3 | 59.2 | 46 |
| **Average Age of Onset** | 46.1 | 51.9 | n/a |
| **Active Disease Visits** | 159 | 13 | n/a |
| **Partial Remission Visits** | 61 | n/a | n/a |
| **Complete Remission Visits** | 174 | n/a | n/a |
| **Ethnicity** |  |  |  |
| **African American** | 12 (4.7%) | 0 (0%) | 14 (6.3%) |
| **Ashkenazi** | 70 (27.7%) | 4 (30.8%) | 46 (20.8%) |
| **Caucasian** | 110 (43.5%) | 4 (30.8%) | 110 (49.8%) |
| **East Asian** | 7 (2.8%) | 2 (15.4%) | 6 (2.7%) |
| **Latino** | 23 (9.1%) | 2 (15.4%) | 10 (4.5%) |
| **South Asian** | 21 (8.3%) | 1 (7.7%) | 18 (8.1%) |
| **Other** | 9 (3.6%) | 0 (0%) | 10 (4.5%) |
| **Unknown** | 1 (0.4%) | 0 (0%) | 7 (3.2%) |
